# Detecting Oral Bacteria in Abdominal Aorta Atherosclerotic Plaques—How Far Can They Go?

**DOI:** 10.3390/medicina61111976

**Published:** 2025-11-04

**Authors:** Aleksandar Šubarić, Zoran Arsić, Đorđe Mihailović, Dragan Marjanović, Vojkan Lazić, Marko Matvijenko, Aleksandra Savić, Marko Stevanović, Danijela Staletović

**Affiliations:** 1 Department of Dentistry, Faculty of Medicine, University of Priština in Kosovska Mitrovica, 38220 Kosovska Mitrovica, Serbiamarko.d.stevanovic@gmail.com (M.S.);; 2 Secondary School “Jovan Cvijić”, 38236 Štrpce, Serbia

**Keywords:** oral microflora, arterial plaques, aneurysm, *Porphyromonas gingivalis*

## Abstract

*Background and objectives*: Atherosclerotic cardiovascular disease (ASCVD) remains a major global cause of mortality, with chronic infections and systemic inflammation, including those arising from periodontal pathogens, contributing to vascular pathology. This study aims to investigate the presence of bacterial DNA from oral cavity microorganisms in atherosclerotic plaques of patients with abdominal aortic aneurysm (AAA) and to explore correlations with oral health indices and clinical variables. *Materials and Methods*: Forty patients (mean age 61 ± 6 years; 60% male) with periodontitis and AAA were included. Subgingival biofilm and arterial plaque samples were analyzed using PCR for *A. actinomycetemcomitans*, *P. gingivalis*, *P. intermedia*, *T. forsythensis*, and *T. denticola*. Dental indices (PI, GI, SBI, and PPD) were determined in all patients, and concordance of the findings was assessed by Cohen’s κ, and correlations were evaluated using Spearman’s coefficient. *Results*: *P. gingivalis* exhibited the highest concordance between oral and arterial plaques, followed by *T. forsythensis* and *A. actinomycetemcomitans*. *T. denticola* DNA was not detected in aortic samples. Positive correlations found between *A. actinomycetemcomitans* and *P. gingivalis* with dental indices were found to be statistically significant. *Conclusions*: The detection of periodontal pathogens, particularly *P. gingivalis* and *T. forsythensis*, in both oral and arterial plaques supports their potential role in atherosclerotic and aneurysmal changes. The findings highlight the relevance of periodontal health in cardiovascular risk prevention and the need for longitudinal studies to reveal causal mechanisms.

## 1. Introduction

Cardiovascular disorders (CVDs), encompassing different entities among which atherosclerotic cardiovascular disease (ASCVD) dominates, are one of the leading causes of death, with around 19 million deaths in 2020 [1]. The cause of CVD has been associated with numerous risk factors, including age, dyslipidemia, metabolic syndrome, hypertension, diabetes, etc. [2,3]. Current studies are showing that there is a connection between infection, systemic inflammation, and immune cross-reactivity with the occurrence of atherosclerotic arterial changes, and finally with CVD [1]. Various types of studies have shown a direct correlation between the presence of oral bacterial colonization, periodontal disease, and atherosclerosis [4]. Interestingly, a recent review suggests that these correlations, although present, need to be further strengthened and examined since the data published are ambiguous [5].

One of the major ASCVDs is rupture of the aortic aneurysm, which has been affected by atherosclerosis [6]. These events are known to correlate with arterial wall inflammatory events, and even therapeutic approaches based on inflammation inhibition have been designed to prevent them potentially [7]. Some mass chronic illnesses (i.e., diabetes) affecting metabolism do tend to contribute to ASCVD by altering the cell structure and response to stress [8]. The stress of the blood vessel can be physical (through changes in blood pressure) and chemical (presence of a pathogen or chemical imbalance in lipid levels), which can lead to the atherosclerotic changes susceptible to further blood vessel damage [9]. Thus, understanding these connections with different risks might help prevent and manage the ASCVD and complications associated with them.

Oral microorganisms, causing various periodontal diseases, colonize the teeth and the gingival margin area. Periodontal diseases are widespread, affecting 20–50% of the world population, with higher prevalence in males [10]. There are hundreds of bacterial species in varying quantities occupying the oral cavity, and their number and diversity depend on oral health [11]. Species associated with periodontal disorder include *Aggregatibacter actinomycetemcomitans*, *Tannerella forsythia*, *Campylobacter rectus*, *Eubacterium nodatum*, *Peptostreptococcus micros*, *Porphyromonas gingivalis*, *Prevotella intermedia*, *Prevotella nigrescens*, *Streptococcus intermedius*, and *Treponema* sp. [11]. Due to their specific features, these bacteria are capable of forming complex structures termed biofilm [12], which in cases of advanced periodontitis can cover large mucosal areas. One of the potential mechanisms through which oral bacteria can exit the oral cavity is through direct invasion of the soft tissue and further spread through the body [13].

With this in mind, this study aims to determine the presence of oral cavity colonizing bacteria DNA in atherosclerotic plaques in the aneurysmal abdominal aorta and compare it with the presence of the same bacterial DNA in the oral cavity. Also, the presence of DNA would be correlated with some oral indices indicative of periodontal diseases and medical history data.

## 2. Materials and Methods

### 2.1. Patients’ Recruitment

In the present study, a total of 40 patients (of both genders) diagnosed with periodontitis and abdominal aorta aneurysm with atherosclerosis were included in the study. The study was conducted at the Clinic for Vascular and Endovascular Surgery of the Clinical Centre of Belgrade, the Dedinje Institute for Cardiovascular Diseases in Belgrade, and the Department of Oral and Dental Diseases at the Military Medical Academy in Belgrade. The Institutional Ethical Committee approved the study protocol (No. 05-1569 approved on 23 September 2015) and all procedures were in accordance with the guidelines and standards given in the Declaration of Helsinki (revised in 2008). All patients enrolled in the study, which was performed from 2015 to 2019, signed a written informed consent prior to the procedures.

### 2.2. Inclusion Criteria

Periodontal disease was diagnosed [14] by an experienced periodontist (D.S.) in patients with at least 4 periodontal pockets exhibiting clinical attachment level (CAL) > 1 mm and periodontal pocket depth (PPD) > 3 mm, located on at least three sites in two different quadrants. According to CAL, patients were classified as those with moderate periodontitis (stage 2) (CAL = 3–4 mm) and severe (stage 3) (CAL ≥ 5 mm). At the same time, atherosclerosis was diagnosed based on the clinical findings, angiography, and Doppler echosonography [15].

### 2.3. Exclusion Criteria

Patients who were receiving antibiotic therapy (presently or in the past three months) or had undergone periodontal therapeutic procedures in the previous three months were excluded from the study. Pregnant women and those with systemic diseases (apart from atherosclerosis) were also excluded from the study.

### 2.4. Periodontal Disease Diagnosis, Clinical Examination, and Dental Indices Determination

Clinical examinations included plaque index (PI), gingival index (GI), sulcus bleeding index (SBI), and periodontal pocket probing depth (PPD), which were determined following standard protocols using the WHO periodontal probe.

The PI (by Silness and Löe) assesses all surfaces of all existing teeth, including vestibular, oral, mesial, and distal [16]. The GI (Löe Silness-GI) is evaluated on all teeth, considering four surfaces: vestibular, oral, mesial, and distal [17].

The Mühlemann–Son SBI evaluates gingival bleeding as an indicator of inflammation [18]. The PPD was measured as the distance from the gingival margin to the bottom of the periodontal pocket, expressed in millimeters [14]. Measurements are taken at six points on each existing tooth on the vestibular (mesial, midpoint of the vestibular surface, and distal) and oral (mesial, midpoint of the oral surface, and distal) surfaces. Periodontal pockets are classified into three groups based on depth to determine the severity of periodontal damage:

1. A depth of 1–3 mm—Normal condition;
2. A depth of 4–6 mm—Stage 1 periodontitis;
3. A depth >6 mm—Stage 2 periodontitis.

### 2.5. Sample Collection

The subgingival biofilm samples were collected using the paper point technique (Periopaper, Amityville, Pro Flow, NY, USA) from the bottom of two out of four present periodontal pockets. Each sample site was isolated with cotton rolls, gently scaled supragingivally, and air-dried. A sterile paper point was inserted into the apical extent of each selected pocket, kept for 60 s, and transferred immediately to a sterile tube. The atherosclerotic plaque samples were obtained during the surgery, during which a sample of atherosclerotic plaque was taken. The method for atherosclerotic plaque sampling was endarterectomy, a standard procedure used for the surgical removal of the arterial plaque. Plaques were stored in the operating room until the end of the operation and were cleaned of any blood contamination with the sterile transport medium. In order to eliminate the blood contamination, the plaque samples were placed in a sterile Eppendorf tube with Tris-EDTA as a transport medium, mixed gently, and stored until DNA extraction.

### 2.6. Sample Preparation

Bacterial DNA was isolated from subgingival biofilm samples by placing paper points in 50 mM NaOH, followed by vortexing and incubation at 95 °C for 5 min. In the case of bacterial DNA isolation from atherosclerotic plaques, the samples were initially incubated in proteinase K solution for 30 min at 56 °C and then for another 15 min at 94 °C in order to inactivate the enzyme. This was performed to fully extract the material from the plaque samples, which are known to be resistant to mechanical and chemical lysis.

After incubation of the mixture, 30 μL of Tris-HCl (pH = 8) solution was added, and the samples were centrifuged for 2 min at 12,100× *g*. The supernatants containing bacterial DNA were transferred to sterile tubes and stored prior to PCR analysis. The DNA recovery from subgingival biofilm ranged from 500 to 1900 ng, while in the case of arterial plaque, DNA recovery ranged from 10 to 180 ng.

### 2.7. PCR Analysis

PCR amplification of 16S ribosomal DNA was used to detect the presence of *P. gingivalis*, *T. forsythensis*, *P. intermedia*, *T. denticola*, and *A. actinomycetemcomitans* in DNA samples isolated from periodontal pockets and atherosclerotic samples. Reactions were carried out in a 25 μL mixture containing 2.5 μL PCR buffer, 2.5 mM MgCl_2_, 0.2 mM dNTPs, 0.2 μM species-specific primers, 1 U DreamTaq DNA polymerase, and 5 μL of bacterial DNA isolate (all reagents from Thermo Fisher Scientific™, Waltham, MA, USA). Positive controls consisted of DNA from reference strains obtained from the American Type Culture Collection (ATCC) given in Table 1. Colonies used for positive controls were cultured, suspended in sterile water, centrifuged, and subjected to DNA extraction, while negative controls contained sterilized distilled water instead of the sample. Amplification was conducted on a thermal cycler (PeqSTAR 2X, PeqLAB Biotechnologie GmbH, Erlangen, Germany) under the following conditions: initial denaturation at 95 °C for 3 min, followed by 35 cycles of denaturation at 94 °C for 45 s, annealing at species-specific temperatures for 60 s, elongation at 72 °C for 60 s, and a final elongation at 72 °C for 5 min. After the terminated reaction, the amount of product was between 10^7^ and 10^9^ copies of the target fragments. Amplified products were resolved by electrophoresis in 8% polyacrylamide gel using 0.5× TAE buffer, stained with ethidium bromide, and visualized under ultraviolet illumination. Primer sequences and corresponding annealing temperatures are given in Table 1.

### 2.8. Statistical Analysis

All data were entered into a database and then used for the statistical program IBM Statistical Package for the Social Sciences version 18.0 (SPSS Inc., Chicago, IL, USA). Continuous variables are expressed as mean ± SD, and categorical variables are reported as a count with percentages. The level of concordance between bacterial DNA presence in biofilms and arterial plaque was assessed by calculating the percentage of observed agreement and Cohen’s kappa coefficient (κ) with corresponding standard errors. The agreement values were interpreted as follows: ≤0.20, slight; 0.21–0.40, fair; 0.41–0.60, moderate; 0.61–0.80, substantial; and ≥0.81, almost perfect agreement [19]. The magnitude of the correlation between the presence of bacterial DNA in arterial plaques or periodontal pockets with sociodemographic characteristics and oral indices was expressed as the Pearson correlation coefficient r and *p* value [20]. The obtained r values were treated as follows: weak (<0.3), moderate (0.3–0.7), and strong (>0.7).

## 3. Results

The group of patients (n = 40) had an average age of 61 years, and males accounted for 60% of the group. Education varied, with 40% having completed only elementary school, 50% completing middle school, and 10% having higher education. Health-related habits include smoking in 60% and alcohol usage in 40%, in addition to endocrine disorders in 40%; high blood pressure was seen in 70% of cases. Regarding family medical history, 70% had cardiovascular disease, 40% had close relatives with periodontitis, while none of their family members had diabetes (Table 2).

Analysis of the presence of bacterial DNA in oral and arterial plaque revealed varying degrees of agreement across species, which is shown in Table 3. The highest concordance (92.5%) with a kappa coefficient of 0.78 ± 0.10 (*p* < 0.001) was found for *P. gingivalis*. Moderate agreement was observed for *T. forsythensis* (80%, κ = 0.54 ± 0.12, *p* < 0.001) and *A. actinomycetemcomitans* (80%, κ = 0.40 ± 0.15, *p* < 0.001), while *P. intermedia* exhibited fair agreement (60%, κ = 0.31 ± 0.09, *p* < 0.001). Interestingly, *T. denticola* DNA was not detected in arterial plaques of examined subjects; thus, the agreement statistics could not be performed.

Spearman’s rank correlation analysis demonstrated several significant associations between the DNA of bacterial strains from arterial plaque and demographic, systemic, and clinical periodontal parameters (Table 4). In the case of *A. actinomycetemcomitans*, significant positive correlations were observed with the gingival index (ρ = 0.542; *p* < 0.001), plaque index (ρ = 0.336; *p* < 0.05), sulcus bleeding index (ρ = 0.477; *p* < 0.01), and probing pocket depth (ρ = 0.453; *p* < 0.05). In contrast, strong negative correlations were identified with education level (ρ = −0.789; *p* < 0.001) and diabetes (ρ = −0.801; *p* < 0.001), and a moderate negative correlation with a family history of cardiovascular disease (ρ = −0.428; *p* < 0.01), indicating higher bacterial prevalence among patients with lower education and without metabolic comorbidities. In the case when the correlation was determined among the presence of *P. gingivalis* in arterial plaque, a moderate-to-strong, positive correlation with oral health indices, particularly the plaque index (ρ = 0.527; *p* < 0.001) and the gingival index (ρ = 0.386; *p* < 0.05), consistent with its established role in periodontal disease progression. Conversely, strong negative correlations were found with endocrine disorders (ρ = −0.667; *p* < 0.001), family history of cardiovascular disease (ρ = −0.801; *p* < 0.001), and diabetes (ρ = −0.667; *p* < 0.001). In contrast, *P. intermedia* presence was strongly associated with education level (ρ = 0.716; *p* < 0.05) and diabetes (ρ = 0.802; *p* < 0.001). However, significant negative correlations were observed with periodontal disease severity markers, including the gingival index (ρ = −0.542; *p* < 0.001), sulcus bleeding index (ρ = −0.477; *p* < 0.001), and probing pocket depth (ρ = −0.453; *p* < 0.001). Finally, the presence of *T. forsythensis* showed weaker correlations overall, with moderate negative associations observed with the gingival index (ρ = −0.453; *p* < 0.01), sulcus bleeding index (ρ = −0.310; *p* < 0.01), and probing depth (ρ = −0.337; *p* < 0.01). A moderate but significant positive correlation was detected with a family history of cardiovascular disease (ρ = 0.356; *p* < 0.01).

## 4. Discussion

The demographic and clinical characteristics of the study group are in accordance with established epidemiological characteristics, which were previously reported. Both age and male gender of the participants are known to be associated with increased risk of abdominal aorta aneurysm [21,22]. Lower educational levels in participants (Table 2) might potentially reflect socioeconomic status and further influence health behavior and access to preventive care [23]. Regarding modifiable risk factors, smoking and alcohol intake are both deemed important for the occurrence of aneurysmal changes [24,25] and were present in higher percentages within the study population.

In all analyzed bacterial species, with measurable DNA, the presence of bacteria was in oral biofilm and aortic plaques, with their occasional absence in arterial plaques (Table 3). These data indicate that the periodontic changes affecting both the tooth and the surrounding tissue enable the systemic spread of these bacteria. This is not the first case pointing to such findings [26] and thus hinting at the potential role of the spread of bacteria in the pathogenesis of arterial plaque formation and, in this case, aneurysmal change in the aorta. Furthermore, it is suggested that periodontitis can be a risk factor for developing atherosclerosis [27], through several mechanisms involving a complex interaction between bacteria and inflammatory response, causing initiation or worsening of atherosclerotic processes.

The potency of specific oral pathogens (*P. gingivalis*, *T. forsythia*, *T. denticola*, and *F*. *nucleatum*) to invade an aortic plaque has been previously proven in animals with increased plasma lipids [5]. The significance of polymicrobial flora has been investigated in vitro, showing that the presence of one bacterial species (*F. nucleatum*) enhances the entry of other species (*P. gingivalis*) into the endothelial cells [28]. These data suggest the potential need for diverse pathogens causing periodontitis to impact atherosclerotic plaque formation and progression directly. In a similar patient population with abdominal aneurysm, a study reported the highest prevalence of *P. gingivalis* and *T. forsythensis* DNA in the examined atheromatous samples [29]. The highest agreement percent, that is, cases where bacteria were present in both oral and arterial plaque, was found for *P. gingivalis* and *T*. *forsythensis* (Table 3). The presence of these two bacteria, together with *T. denticola*, is termed a red complex, which is basically an aggregate (biofilm) associated with severe clinical presentation of periodontal disease [30]. Although periodontitis is regarded as a prototype of chronic infection of low intensity, it is connected with a medium-intensity systemic inflammatory response [31].

Some of the examined bacterial species, such as *P. intermedia*, serve as a primary colonizer of the oral cavity and create an environment for secondary colonizers such as *P. gingivalis* [32]. Numerous and extensive studies show that *P. gingivalis* enters the bloodstream via direct invasion through oral ulcerations or lymphatic vessels [33]. The same ability to enter the bloodstream and invade blood vessels has been shown for *T. forsythensis* as well [34,35]. When they reach the endothelial cells, they promote oxidative stress and inflammation, activated through the interaction between cell signaling cascades and molecules located on the bacterial wall [36]. Contributing to the formation of atherosclerosis should also be attributed to *P. gingivalis*’ ability to promote foam cell formation directly [37] and that of the *T. forsythensis* to increase the atherogenic potency of low-density lipoproteins [38]. One of the potential reasons for the absence of *P. intermedia* and *T. denticola* in arterial plaque might be their poor ability to form biofilm on their own, or to affect immune system response via different virulence factors [30].

These bacteria also cause alterations in vascular smooth muscle cells, thus potentially contributing to the aneurysm changes in the abdominal aorta. Understanding this mechanism is important since the samples collected for the present study are from patients with aneurysmal changes in the abdominal aorta. These often-fatal aneurysmal changes are associated with plaque buildup and plaque progression, causing intense oxidative stress, matrix degradation, and apoptosis of smooth-muscle cells [6]. The presence of *P. gingivalis* has been known to impact the migration and proliferation of vascular smooth muscle cells and, at the same time, cause a reduction in expression and activity of tissue factor inhibitor [39]. Furthermore, the final process in the atherosclerotic alteration of the blood vessel, calcification, has been found to be highly influenced by the *P. gingivalis* [36].

**Figure 1:**
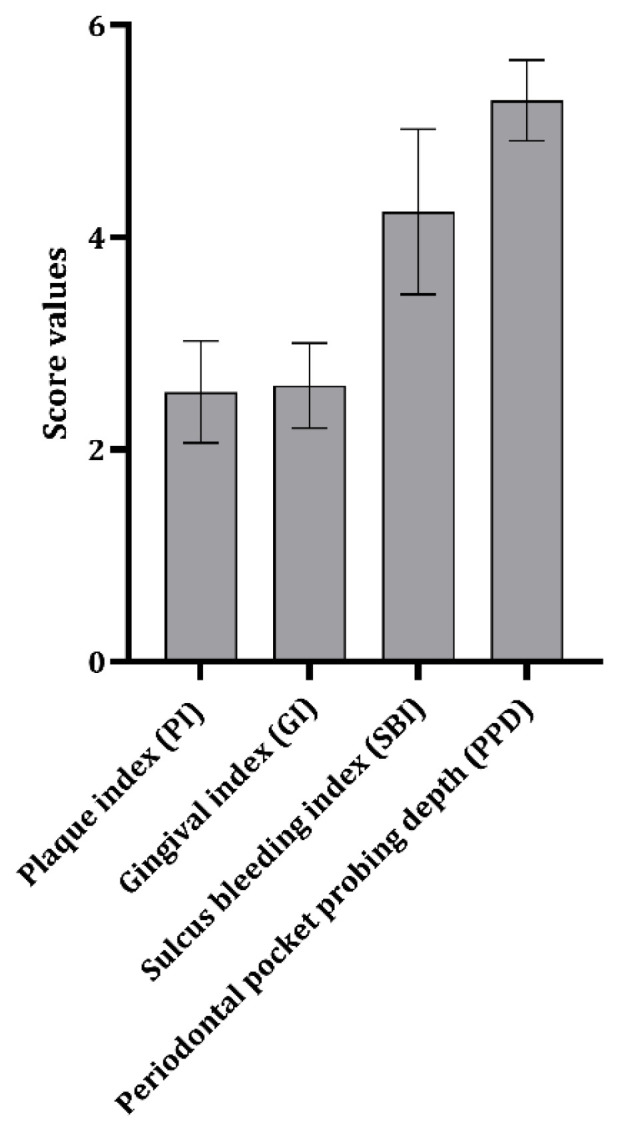
Dental indices in patients with abdominal aorta aneurysms.

The clinical picture of periodontitis varies from persistent gingivitis to certain forms of extremely destructive periodontitis affecting different tissue structures. Biofilms, dominantly that of subgingival localization, are the main etiological factor for periodontitis [31], and the indices used in the present study are partially used to estimate the extent of biofilm formation (Figure 1) and the clinical picture of periodontitis. The presence of *P. gingivalis* in arterial plaque and reactivity of the gingival tissue, estimated through indices, has been found to correlate moderately (Table 4). On the other hand, the presence of *P. intermedia* in arterial plaques negatively correlated with oral indices (Table 4). Thus, these data indicate that the usage of such indices might not be entirely indicative of the presence of the bacteria in plaques. This being said, it is speculative whether the bacterial invasion of arterial plaque occurred recently and is connected to the current oral microflora or not. Also, one cannot fully exclude the usage of antibiotics in patients since their check was carried out based on available medical records and patients’ self-check questionnaires. Another potential contributor is the change in oral microbiota occurring during periodontitis. Future longitudinal and follow-up studies might focus on this result.

A recent large metagenomic study highlighted the potential role of relatively common bacterial species in the occurrence and progression of CVD. These include bacteria from the genus *Escherichia*, *Prevotella*, *Bacteroidetes*, *Lactobacillus*, *Ruminococcus*, *Eubacterium*, and *Streptococcus* [1]. One of them has been investigated in the present study, and its presence in AAA has been confirmed. The presence of these bacteria correlated positively with both previous periodontitis and cardiovascular events in family history (Table 4), which further confirms the claims of this study. There is also a moderate correlation with endocrine disorders (Table 4), which might hint toward another role of these bacteria.

Both periodontitis and atherosclerosis are known to share common risk factors such as smoking, diabetes mellitus, obesity, etc. [40]. The aforementioned common risk factors are known to cause oxidative stress and alter body metabolism, and it is not surprising that they contribute to different disease progression [6]. Interestingly, the presence of the bacterial DNA in arterial plaques mostly negatively correlated with the examined risk factors for CVD, suggesting that their presence might not be associated with the presence of bacteria in plaque samples (Table 4). These conclusions are based on a limited sample (n = 40) of arterial plaques obtained from abdominal aortic aneurysms; thus, one might associate the findings with this type of CVD.

This study has certain strengths, but also limitations. The main strength of the study is the specific oral bacteria DNA that were examined using a sensitive technique. Also, the findings of the study corroborated some previous data regarding risk factors and pathogenic mechanisms, thus reinforcing the link between periodontitis and CVD. The limitations of the study are the type of study (cross-sectional) and the limited sample size, even though the subjects met specific inclusion and exclusion criteria. One might argue that the examined bacteria panel should potentially be broader, thus enabling data interpretation through a different lens.

## 5. Conclusions

This study demonstrates a notable presence of periodontal pathogens, particularly *P. gingivalis* and *T. forsythensis*, within both oral and arterial plaques of patients with abdominal aortic aneurysms, supporting their previously hypothesized potential role in the pathogenesis of atherosclerotic and aneurysmal changes. Strong concordance between oral and arterial samples for these species highlights their ability to disseminate systemically and colonize vascular tissues. Correlations between bacterial presence and clinical periodontal indices suggest that disease severity in the oral cavity may contribute to vascular colonization and reinforce the importance of periodontal health in patients with ASCVD. Future longitudinal studies to clarify causal mechanisms and assess the potential benefits of periodontal therapy in mitigating vascular disease progression are needed to confirm these hypotheses.

## Figures and Tables

**Table 1:** Primers used for PCR, their hybridization temperatures, and product sizes, as well as ATCC code for positive controls.

Bacterial Species	Sequence (5′-3′)	Hybridization Temperature (°C)	Product Size (bp)	ATCC
*A. actinomycetemcomitans*	Fwd GCTAATACCGCGTAGAGTCGG Rv ATTTCACACCTCACTTAAAGGT	55	500	33384
*P. gingivalis*	Fwd AGGCAGCTTGCCATACTGCG Rv ACTGTTAGCAACTACCGATGT	55	400	33277
*P. intermedia*	Fwd CGTGGACCAAAGATTCATCGGTGGA Rv CCGCTTTACTCCCCAACAAA	55	259	33563
*T. forsythensis*	Fwd GCGTATGTAACCTGCCCGCA Rv TGCTTCAGTGTCAGTTATACCT	55	600	43037
*T. denticola*	Fwd TAATACCGAATGTGCTCATTTACAT Rv TCAAAGAAGCATTCCCTCTTCTTCTTA	60	316	35405

**Table 2:** Patient profile depending on the plaque sample collection artery.

Number of patients (n)	40
Age (mean ± SD)	61 ± 6
Gender, male (%)	24 (60)
Education level (%)	
Elementary	16 (40)
Middle	20 (50)
Higher	4 (10)
*CV risks*	
Smoking (%)	24 (60)
Alcohol consumption (%)	16 (40)
Endocrine disorders (%)	16 (40)
Increased blood pressure (%)	28 (70)
*Family medical history*	
Cardiovascular	28 (70)
Periodontitis	16 (40)
Diabetes	0 (0)

Estimated dental indices were around 2 for PI and GI, around 4 for SBI, and around 5 for PPD (Figure 1).

**Table 3:** Overlap between the presence of bacterial DNA in the abdominal aorta and biofilms.

Bacterial Species		Biofilms		Statistical Parameters		
	Arterial Plaque	Yes	No	% of Agreement	Kappa ± SE	*p*-Value
*A. actinomycetemcomitans*	Yes	4	0	80	0.4 ± 0.15	<0.001
	No	8	28			
*P. gingivalis*	Yes	24	0	92.5	0.78 ± 0.1	<0.001
	No	4	12			
*P. intermedia*	Yes	12	0	60	0.31 ± 0.09	<0.001
	No	16	12			
*T. forsythensis*	Yes	24	0	80	0.54 ± 0.12	<0.001
	No	8	8			
*T. denticola*	Yes	0	0	NA	NA	NA
	No	16	24			

NA—not applicable.

**Table 4:** Correlation analysis between the presence of bacterial DNA in aorta plaque and sociodemographic characteristics or oral indices.

Bacterial Strain	*A. actinomycetemcomitans*	*P. gingivalis*	*P. intermedia*	*T. forsythensis*
Parameter	Spearman’s coefficient; *p*-value	Spearman’s coefficient; *p*-value	Spearman’s coefficient; *p*-value	Spearman’s coefficient; *p*-value
Gender	0.356; <0.05	0.250; >0.05	−0.354; <0.05	−0.250; >0.05
Education level	−0.789; <0.001	−0.389; <0.05	0.716; <0.05	−0.640; >0.05
*Cardiovascular risks*				
Smoking	−0.089; >0.05	−0.167; >0.05	0.089; >0.05	−0.250; >0.05
Alcohol consumption	0.081; >0.05	−0.251; >0.05	−0.089; >0.05	0.250; >0.05
Endocrine disorders	−0.356; <0.05	−0.667; <0.001	0.356; >0.05	0.250; >0.05
Increased blood pressure	0.047; >0.05	−0.356; <0.05	−0.048; >0.05	−0.089; >0.05
*Family history*				
Cardiovascular	−0.428; <0.01	−0.801; <0.001	0.429; <0.001	0.356; <0.01
Periodontitis	−0.534; <0.001	−0.583; <0.001	0.535; <0.001	0.167; >0.05
Diabetes	−0.801; <0.001	−0.667; <0.001	0.802; <0.001	0.275; >0.05
*Oral indices*				
Plaque index	0.336; <0.05	0.527; <0.001	−0.337; <0.001	−0.100; >0.05
Gingival index	0.542; <0.001	0.386; <0.05	−0.542; <0.001	−0.453; <0.01
Sulcus bleeding index	0.477; <0.01	0.094; >0.05	−0.477; <0.001	−0.310; <0.01
Periodontal pocket probing depth	0.453; <0.05	0.219; >0.05	−0.453; <0.001	−0.337; <0.01

Correlation with the presence of *T. denticola* was not possible since there were no cases with bacterial DNA in the arterial plaque.

## Data Availability

Data will be available upon reasonable request from the corresponding author.

## Abbreviations

The following abbreviations are used in this manuscript:

AAA	Abdominal aortic aneurysm
ASCVD	Atherosclerotic cardiovascular disease
CAL	Clinical attachment level
CVD	Cardiovascular disorders
GI	Gingival index
PI	Plaque index
PPD	Periodontal pocket depth
SBI	Sulcus bleeding index
